# Seasonal variation in gut microbiota of migratory wild raptors: a case study in white-tailed eagles

**DOI:** 10.1186/s42523-025-00406-y

**Published:** 2025-04-17

**Authors:** Xiaoqi Ouyang, Yu Guan, Jianchi Pei, Jianping Ge, Hongfang Wang, Lei Bao

**Affiliations:** 1 Ministry of Education Key Laboratory for Biodiversity Science and Ecological Engineering, College of Life Sciences, Beijing Normal University, Beijing, 100875 China; 2 National Forestry and Grassland Administration Key Laboratory for Conservation Ecology of Northeast Tiger and Leopard, Beijing, 100875 China

**Keywords:** White-tailed eagle, Gut Microbiome (microbiota), Seasonal change, Pathogen, Migratory raptors, 16S rRNA gene

## Abstract

**Background:**

Migration poses significant energetic challenges for migratory birds, during which both intrinsic and extrinsic factors affecting the gut microbiota alter substantially. While the temporal dynamics of gut microbiota in wild birds across migration seasons have garnered increasing attention, research on the seasonal variation in wild raptors remains limited despite their distinct gut microbiota structures. Furthermore, raptors, being the highest trophic level in the food chain, have been found to harbor more pathogens and antibiotic resistance genes (ARGs). In this study, we characterized the diversity and composition of the gut microbiota of wild white-tailed eagles at a critical stopover site along the East Asian Flyway (EAF). Fecal samples were collected during both autumn and spring migration seasons and microbial compositions were analyzed using high-throughput sequencing.

**Results:**

The most prevalent bacterial phylum in the gut microbiome of white-tailed eagles during both migration seasons was Firmicutes. The diversity of the gut microbiota is elevated in the spring migration season and the bacterial community composition significantly differed between two seasons. Individuals in spring migration show elevated levels of *Clostridium_sensu_stricto_13* and *Brochothrix*, most likely related to the consumption of carrion. Conversely, individuals in autumn migration showed a higher prevalence of potential pathogens such as *Fusobacterium* and *Escherichia-Shigella*. Furthermore, we found that specific genera were seasonally enriched, probably reflecting distinct environmental exposures along migration routes.

**Conclusions:**

This study revealed substantial seasonal variation in the gut microbiota of migratory white-tailed eagles, most likely shaped by dietary shifts, environmental factors, and physiological stress during migration. The higher prevalence of pathogens during autumn migration highlights potential health risks for eagles and their ecosystems, emphasizing the need for targeted conservation strategies at stopover sites. These findings contribute to understanding the dynamic interactions between migration and gut microbiota in wild raptors and provide valuable insights into their ecological and health management. While dietary differences may play a role, further research is needed to directly assess their impact.

**Supplementary Information:**

The online version contains supplementary material available at 10.1186/s42523-025-00406-y.

## Introduction

The gut microbiota is known to affect numerous host-associated functions including dietary specialization [1], stimulation of immune system [2], metabolic capacity [3] and competitive exclusion of pathogens [4]. The composition of the gut microbiota exhibits high variability, changing in response to both internal (e.g., age, sex, physiological state, reproductive patterns, host genetics) and external (e.g., environment, diet, location, temperature, social interactions) factors [5–7]. Unlike other vertebrates, birds exhibit substantial intraspecific variation in their gut microbiota [8], which may be attributed to their unique life cycle, diet, and ecological factors [8–10]. Consequently, there has been considerable interest in understanding how these factors influence the variation of gut microbiota within host species.

Migration, a unique life cycle， provides an intriguing scenario for studying temporal patterns and the influence of environment and diet on the gut microbiota of wild birds [6, 11]. Every year, billions of migratory birds travel thousands of kilometers between breeding and non-breeding habitats to take advantage of seasonal fluctuations in resource availability [12]. Long-distance migration is an energetically demanding process, promoting migratory birds to develop a range of physiological adaptations that optimize their performance during migration [13, 14]. For instance, digestive organs including gizzard, small intestine and colon may be remodeled in size to meet the fluctuating demands of flight, which in turn alters the physical habitat of the gut microorganisms. In addition to these intrinsic physiological adjustments, migratory birds encounter significantly different environmental conditions at their wintering grounds and one or more stopovers [15], which may contribute to shifts in their gut microbiota. Furthermore, many species of migratory birds have evolved to adjust to seasonal dietary changes, enabling them to exploit periods of food abundance [16]. For example, swan geese (*Anser cygnoides*) shift from feeding on aquatic vegetation in wintering grounds to agricultural crops in stopover sites [17], while red knots (*Calidris canutus*) adjust their digestive organ size seasonally to optimize the digestion of different prey types encountered along their migratory route [18].

Given the intrinsic physiological adaptations and extrinsic dietary and environmental shifts during migration, the diversity and composition are also expected to be responsive to migration. Recent studies have investigated how environmental factors and movement patterns can affect the microbiome of different migratory wild bird species. For example, microbial communities have been shown to vary between wintering and breeding grounds within the same population in Kirtland’s Warblers (*Setophaga kirtlandii*) [19] and swan geese [20], as well as between migration seasons in passerines [21] and Hooded Crane (*Grus monacha*) [22]. Furthermore, a previous study has found that a broader range of activity is significantly linked to higher microbiota diversity, likely due to increased exposure to diverse environmental conditions [23]. Additionally, gut microbiota can rapidly adapt to local environmental microbial communities, with longer durations spent at stopover sites in passerines leading to greater convergence between the host microbiota and the local microbial pool [6, 22]. However, contrary to previous studies, Risely et al. [24] found no significant difference in gut microbiota between migratory and resident Red-necked stints (*Calidris ruficollis*) and that most individuals source as little as 0.1% of gut microbes from their environment. Therefore, further research is required to assess whether the higher resistance to environmental microbiota is general across migratory species.

Raptors, as obligate carnivores, possess a distinct dietary niche compared to other avian ecological groups, which contributes to their unique gut microbiota composition and bacterial functional potential [25]. However, despite these notable differences, no study to date has systematically investigated the seasonal variation in the gut microbiota of wild migratory raptors, highlighting a critical gap in our understanding of host-microbe interactions in these top predators.

The white-tailed eagle (*Haliaeetus albicilla*) is an apex predator with a wide distribution across northern Eurasia, spanning from Greenland and Iceland in the west to Japan in the east, and extending as far south as North Africa [26]. The species follows well-defined migration routes along the East Asian-Australasian Flyway (EAAF), with breeding populations primarily located in Russia, Mongolia, and northeastern China, and wintering populations concentrated in the Yangtze River Basin, Japan, and the Korean Peninsula. The species inhabits diverse aquatic ecosystems, including coastal wetlands, large rivers, and lakes, which provide abundant food resources [27]. Seasonal variation in habitat use is largely driven by food availability and climatic conditions. During migration and wintering periods, white-tailed eagles rely on stopover wetlands, such as the Jingxin Wetland, for foraging and resting. The diet of white-tailed eagles varies significantly across seasons and regions. They primarily consume fish, waterfowl, and small mammals, but during food shortages, they scavenge carrion, particularly in winter [28]. This seasonal dietary shift may play a crucial role in shaping gut microbiota composition, particularly by increasing exposure to environmental microbes from carrion [29].

Jingxin Wetland, a key stopover site along the EAAF [26], supports one of the largest known congregations of white-tailed eagles during migration. This makes it an ideal location for investigating how migration influences gut microbiota composition in large raptors. Recently, the gut microbiota of wild birds has received widespread attention also for serving as a primary source of zoonotic pathogens and antibiotic resistance genes (ARGs) that contribute to human and animal diseases [30, 31]. Migratory birds, by traversing diverse regions annually, may facilitate the acquisition and global dissemination of pathogens [32, 33]. Notably, studies have suggested that pathogen prevalence and ARGs tend to increase with trophic level, placing apex predators such as raptors at a heightened risk [31].

In this study, we collected fecal samples during the autumn and spring migration seasons at the Jingxin Wetland to compare seasonal changes in the gut microbiota of white-tailed eagles. Our goals were to: (1) characterize and compare the diversity and composition in the gut microbiota of white-tailed eagles at two distinct periods of the annual cycle; (2) determine if potential pathogens are present and analyze their seasonal variations in abundance.

## Results

### Overview

**Fig. 1:**
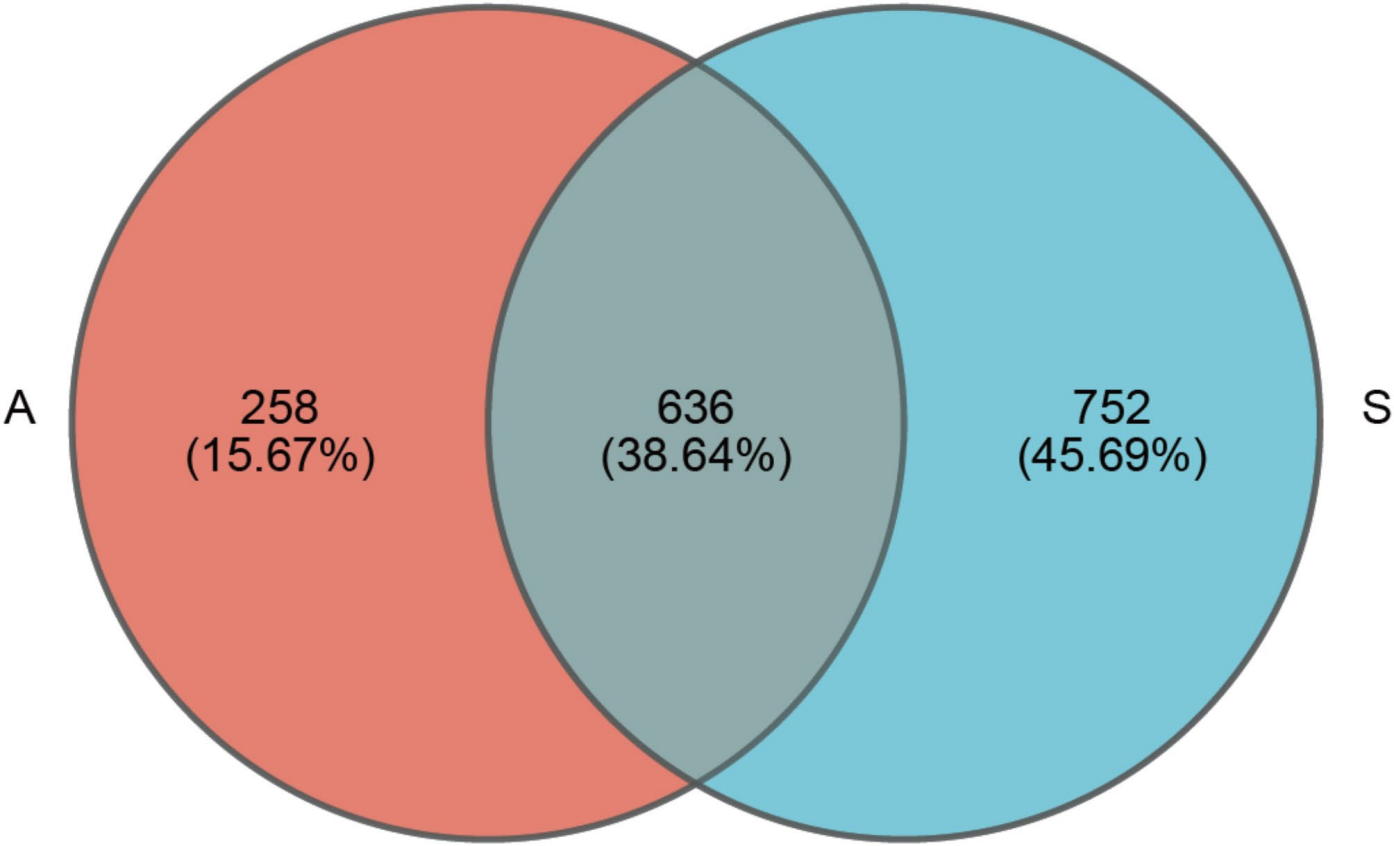
Venn diagram showing the unique and shared intestinal bacterial operational taxonomic units (OTUs) of white-tailed eagles between the autumn (**A**) and spring (**S**) seasons

We obtained 2,342,587 high-quality reads from the raw sequencing data after quality processing, ranging from 36,734 to 138,008 (mean = 70,987). In total, 1,646 OTUs were obtained with a 97% similarity threshold from 33 fecal samples, 38.64% of which were identified in both seasons. The number of unique gut bacterial OTUs were 258 (15.67%) and 752 (45.69%) in autumn and spring, respectively (Fig. 1). Rarefaction curves of all 33 tended to be saturated (Fig. S1), indicating the sufficient sequencing depths (36,267 reads) for further analysis. The gut microbiota of white-tailed eagles was primarily composed of Firmicutes, followed by Proteobacteria, Actinobacteriota, and Fusobacteriota, with seasonal differences in their relative abundances. At the genus level, *Clostridium_sensu_stricto* and *Megamonas* were dominant in both seasons, while certain taxa varied between autumn and spring.

### Alpha diversity

**Fig. 2:**
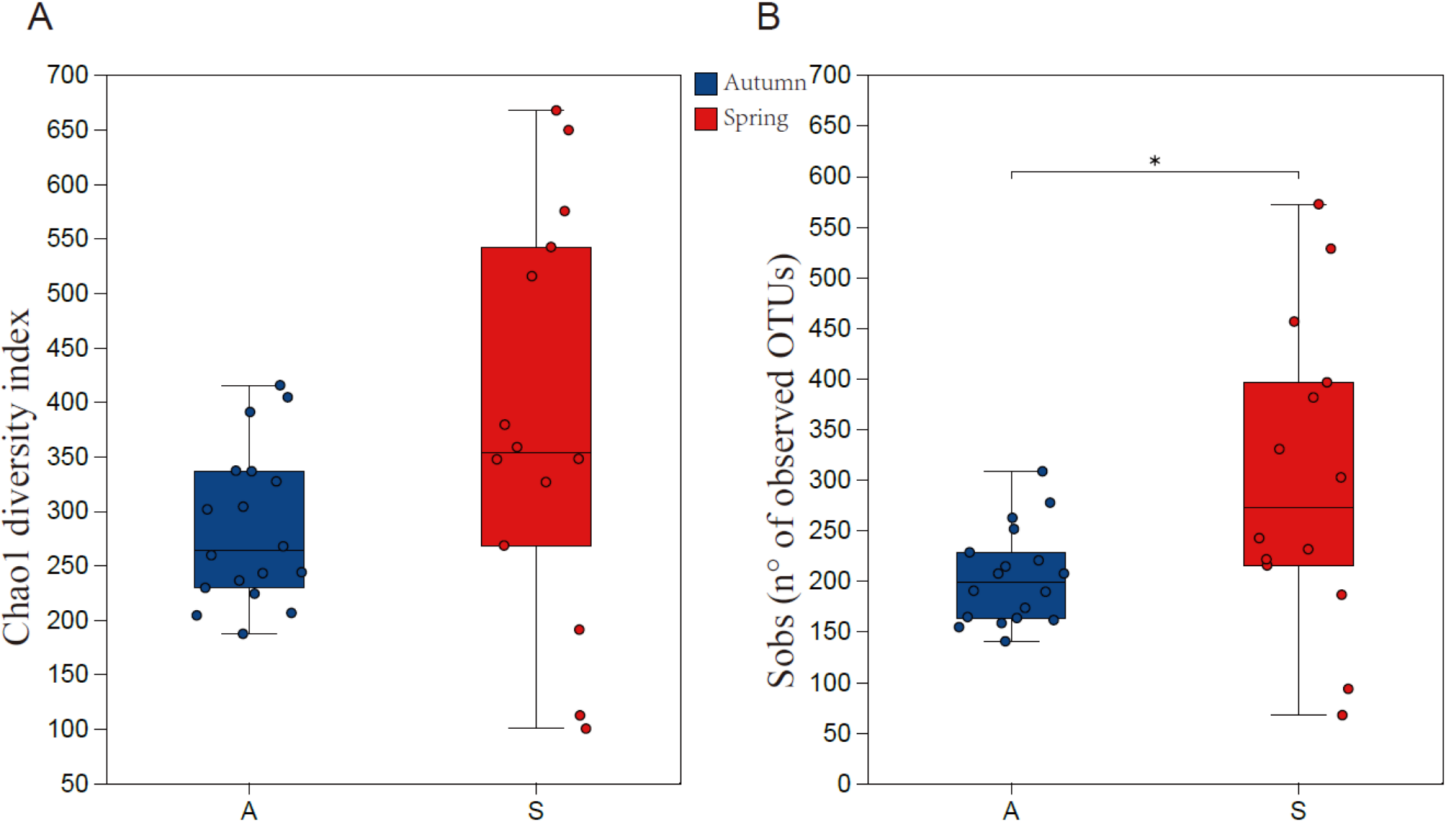
The Wilcoxon rank-sum test for alpha diversity indices of white-tailed eagle samples, including (**A**) Chao1and (**B**) Sobs indices. A means the autumn migration group, while S means spring migration group

Although not statistically significant across all metrics, alpha diversity tended to be higher during the spring migration season, as indicated by the Sobs (the observed richness) (*p*  < 0.05) and Chao1 (*p* = 0.077) indices (Wilcoxon rank-sum test) (Fig. 2).

**Fig. 3:**
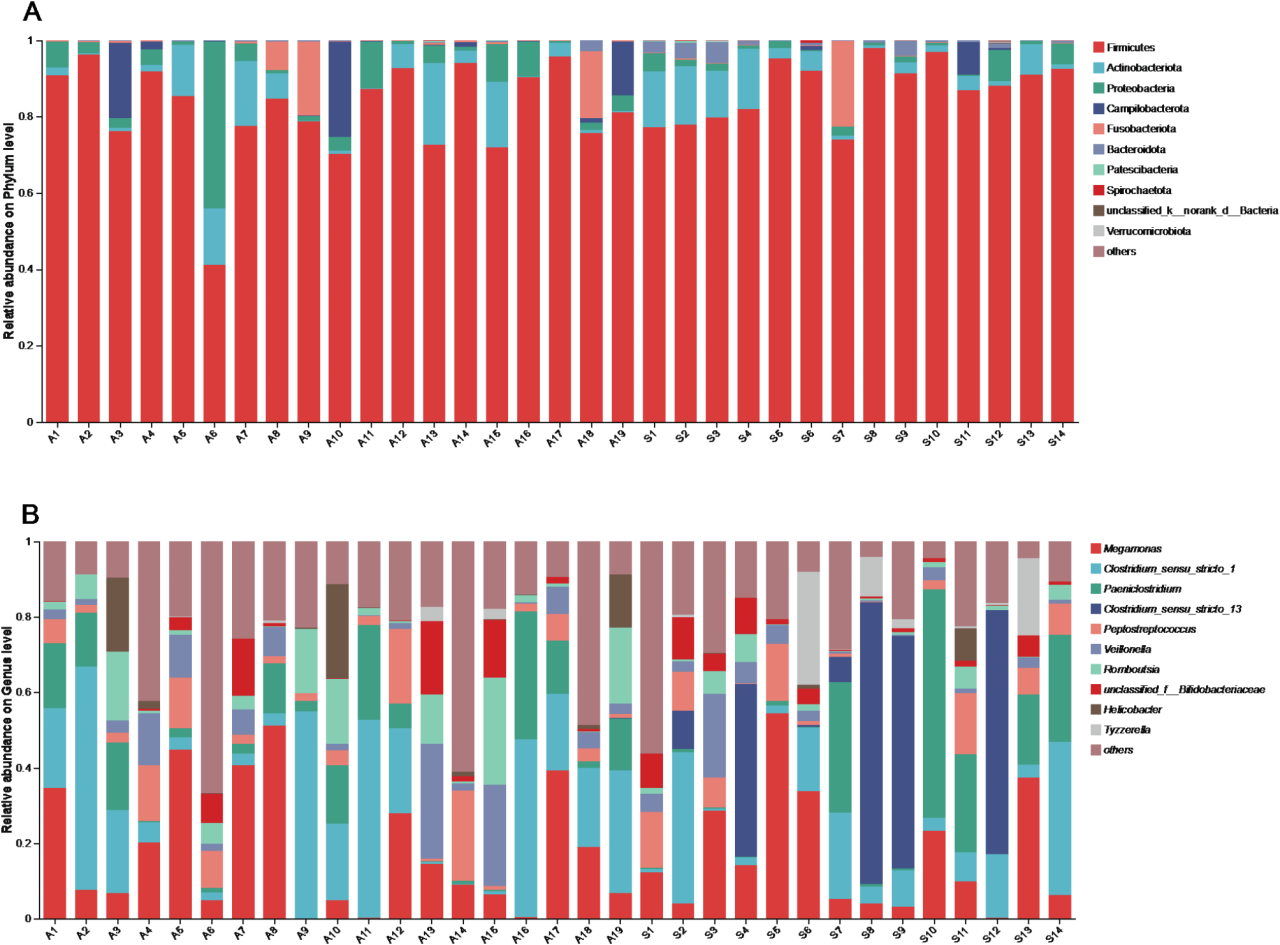
Composition of gut microbiotas in the phylum (**A**) and genus (**B**) level of two migration seasons. Columns marked “A” represent the autumn migration, and columns marked “S” represent the spring migration season

A total of 30 phyla, 77 classes, 169 orders, 291 families, and 634 genera were detected (Fig. 3). In the autumn group, Firmicutes (81.80%) was the most predominant phylum, followed by Proteobacteria (6.04%), Actinobacteriota (5.85%), Campilobacterota (3.39%) and Fusobacteriota (2.55%) (Fig. S2A). At the genus level, *Clostridium_sensu_stricto_1* (19.62%), *Megamonas* (18.30%), *Paeniclostridium* (9.25%), *Romboutsia* (7.83%) and *Veillonella* (6.74%) were the top five genera in the autumn migration group (Fig. S2B).

In spring migratory eagles, Firmicutes (87.31%) remained the most dominant phylum while Actinobacteria (6.13%), Proteobacteria (2.26%), Fusobacteriota (1.67%) and Bacteroidota (1.55%) ranked from second to fifth (Fig. S2A). The genus *Clostridium_sensu_stricto_13* (18.81%) was the most abundant, followed by *Megamonas* (16.29%), *Paeniclostridium* (12.29%), *Clostridium_sensu_stricto_1* (12.26%) and *Peptostreptococcus* (6.07%) (Fig. S2B).

### Beta diversity

**Fig. 4:**
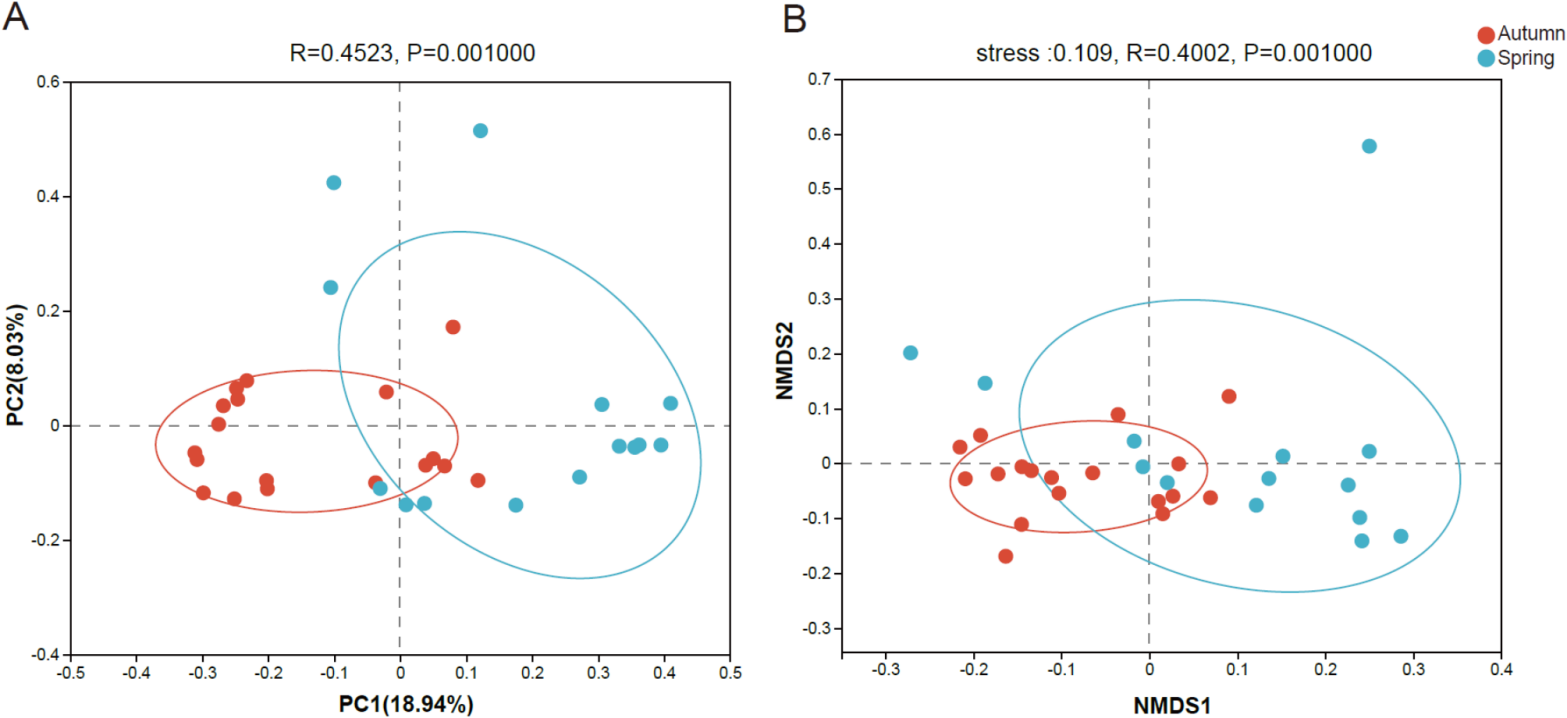
Principal Coordinate Analysis (PCoA) (**A**) and Non-Metric Multidimensional Scaling (NMDS) (**B**) plots of gut microbiota composition in white-tailed eagles during autumn and spring migrations. PCoA and NMDS were generated with Binary Jaccard distances and unweighted UniFrac distances respectively. A means autumn migration group while S represents spring migration group

Community-level analysis revealed sharp distinctions in the beta diversity of gut microbiota in white-tailed eagles between different migration seasons, when comparing across the full data set with both Binary Jaccard distances (*R* = 0.452, *p* = 0.001) and unweighted Unifrac distances (*R* = 0.400, *p* = 0.001) (Fig. 4).

### Differentially abundant taxa

**Fig. 5:**
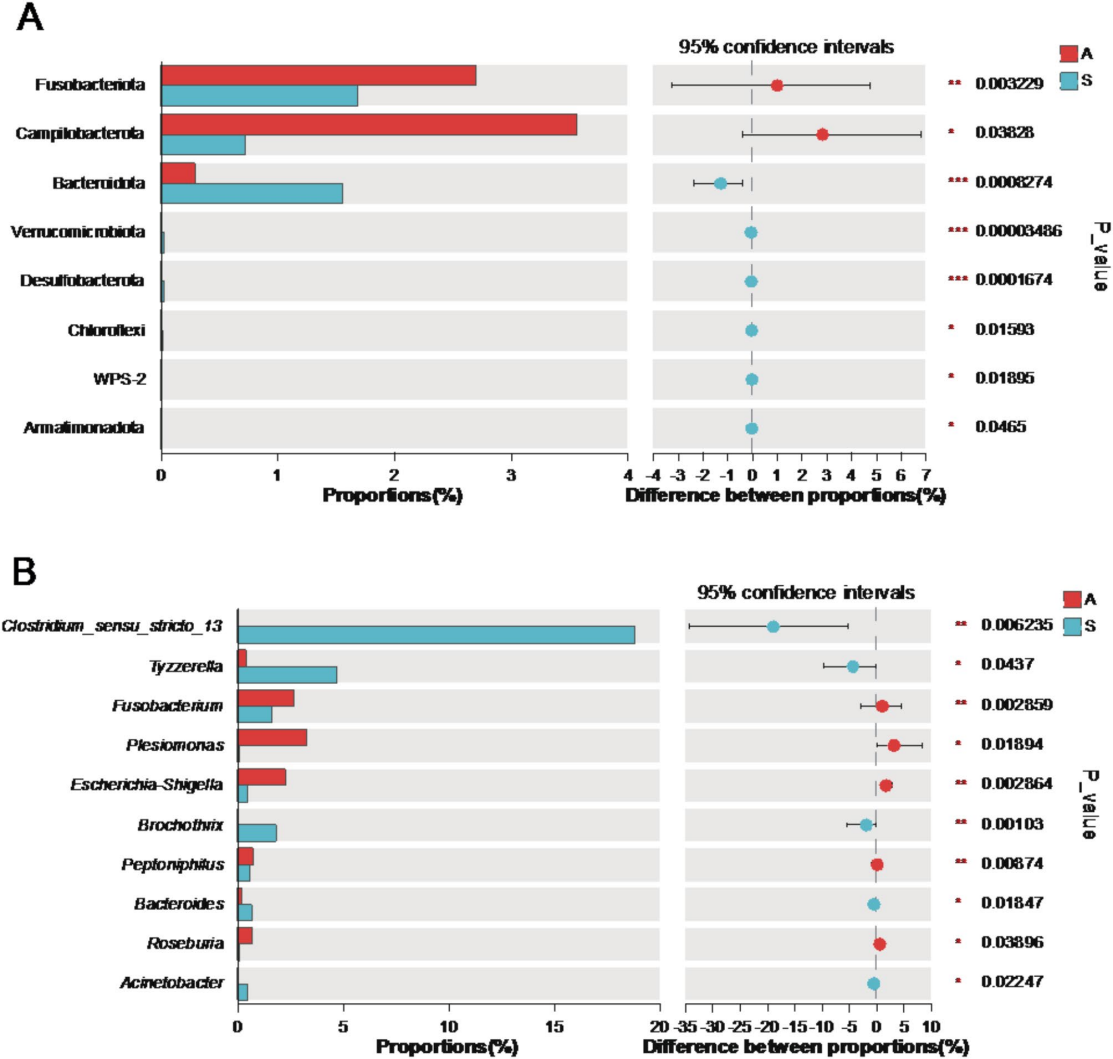
Wilcoxon rank-sum test bar plot at phylum (**A**) and genus (**B**) level. The X-axis (left panel) represents the relative proportions (%) of microbial taxa in different groups, with bars of different colors representing different groups. The Y-axis represents the relative abundance of microbial taxa at the phylum (**A**) and genus (**B**) levels. A refers to the autumn migration group, while S represents the spring migration group. The right panel displays the difference in proportions between groups with 95% confidence intervals. Significance levels are indicated as follows: One asterisk (*), two asterisks (**) and three asterisks (***) indicate a *p*-value less than 0.05, 0.01 and 0.001, respectively

We determined bacterial phyla and genera that exhibited significant variation in abundance between different migration seasons using the Wilcoxon rank-sum test. A total of 8 phyla and 10 genera displayed significant different abundance between seasons (Fig. 5). In particular, the relative abundance of genus *Clostridium_sensu_stricto_13*, *Brochothrix*, *Bacteroides* and *Acinetobacter* was significantly elevated in the spring migration season compared to the autumn migration. The autumn migration group exhibited a higher relative abundance of the phylum Campylobacterota and the genera *Fusobacterium*, *Escherichia-Shigella*, and *Plesiomonas* compared to the spring migration group. Notably, Campylobacterota, *Fusobacterium*, and *Escherichia-Shigella* include potential pathogens carried by migratory birds.

## Discussion

In this study, we collected a total of 33 gut microbiota samples from white-tailed eagles, with 17 samples from the autumn migration and 14 from the spring migration. Our findings revealed notable seasonal differences in microbiota diversity and composition. This study highlights the dynamic nature of gut microbiota in migratory raptors and raises important questions about the role of migration, diet, and environment in shaping microbial communities.

### Community composition

Firmicutes were the predominant phylum in both the autumn and spring migration seasons, consistent with previous findings in various avian species, including chickens [34], gentoo penguins (*Pygoscelis papua*) [35], *Procellariiform* seabirds [36], hooded cranes [22, 37] and swan geese [20]. In white-tailed eagles, the average proportion of Firmicutes exceeded 80% in both seasons, surpassing the levels reported in the aforementioned species. Compared to the captive white-tailed eagles in Japan, whose gut microbiota was primarily dominated by Firmicutes and Proteobacteria [38], the gut microbiota of white-tailed eagles during autumn migration exhibited greater similarity to this composition, while during spring migration, the dominant phyla were Firmicutes and Actinobacteria. Members of the phylum Firmicutes are known for their role in breaking down complex carbohydrates, polysaccharides, sugars, and fatty acids, which are major nutrient sources for all animal hosts [39, 40].

Similar phylum-level patterns have also been reported in other raptor species. For instance, in common kestrels (*Falco tinnunculus*), Firmicutes and Proteobacteria were identified as dominant phyla, and notable shifts in gut microbial composition were observed between healthy and injured individuals, potentially influenced by physiological or environmental factors [41]. Moreover, a comparative study on wild and captive raptors found that while Firmicutes and Bacteroidota predominated across groups, wild individuals harbored higher microbial diversity, suggesting that captivity, diet, and environmental exposure strongly affect gut microbiota structure [42].

The relative abundances of all detected phyla exhibited notable seasonal variation, sometimes changing dramatically. Seasonal shifts in major bacterial taxa have been previously documented in migratory birds across their annual cycles [19, 43, 44]. This study, however, is the first to explore such shifts in raptors across different migration seasons. Variation in microbiome composition across the migration seasons may reflect differences in migration behavior, presence or abundance of environmental bacteria [20] and/or dietary changes [45] that in turn favor some bacteria over others or adapt to the host’s physiological needs [44]. Below, we consider plausible examples of each.

### Migration

Long-distance migration, characterized by intense physical demands [41, 42, 46, 47], which is associated with decreased immune function [48, 49], has been suggested to increase pathogen susceptibility of the migrants [7, 21]. In addition, hosts that move between regions are naturally exposed to parasites in each of the regions they visit, resulting in migratory species experiencing a higher diversity of parasite exposure and infection [50]. While pathogenicity was not directly assessed in this study, we observed an increased relative abundance of several bacterial genera that contain known pathogenenic strains in autumn migration season.

In particular, *Fusobacterium*, and *Escherichia-Shigella* were significantly enriched in the autumn migration season when compared to the spring migration group. *Fusobacterium nucleatum*, belonging to genus *Fusobacterium*, is a critical member of both the oral and gut microbiotas. Increased abundance of *F. nucleatum* is reported to be associated with several human diseases, including oral infections and gastrointestinal diseases such as inflammatory bowel disease (IBD) [51]. Moreover, *Esherichia-Shigella*, with the potential to disrupt intestinal ecological balance, exacerbate intestinal damage, and prolong inflammatory responses in humans [52], also serves as primary pathogen carried by wild birds [31, 53]. The Jingxin Wetland is situated at the southern edge of the breeding range and the northern limit of the wintering range for white-tailed eagles in Asia, suggesting that they likely undergo longer migratory distances before reaching the wetland during autumn migration. The greater physical stress caused by these extended migratory distances may make white-tailed eagles more susceptible to infection by pathogens. Consequently, elevated levels of potential pathogens were detected in white-tailed eagles during the autumn migration.

Special attention should be paid to migratory raptors, as Liu et al. [31] have recently reported that carnivorous migratory birds at higher trophic levels harbored abundant pathogens, such as Shigella and high-risk ARGs. As apex raptors, white-tailed eagles were found to carry high level of pathogens like *Escherichia-Shigella and Fusobacterium.* These pathogens are likely to be disseminated along their migration routes and may pose a threat to human health since many of the pathogens are zoonotic [54]. For example, *Escherichia coli*, belonging to genus *Escherichia-Shigella*, was found to be significantly abundant during the autumn migration of white-tailed eagles. This bacterium has been shown to be indirectly transmitted to humans via migratory birds [30]. Therefore, it is essential to recognize the significant role that migratory raptors play in the transmission of pathogens responsible for various human and animal diseases, supporting the development of fact-based precautionary measures.

Prior studies have indicated that, compared with the non-migratory birds, the abundance of the genus *Corynebacterium* increases [54, 55], likely due to its role in promoting fat deposition or immune responses induced by the stress of migration [24]. However, in this study, this genus was detected at negligible levels in white-tailed eagles during both the autumn and spring migration (autumn: 0.1%, spring: 0.15%), contrary to the reported studies (e.g., 23% in migrating *Calidris ruficollis*). This finding suggest that the role of *Corynebacterium* varies across host species and further research on its function is required.

### Environmental effect

The avian gut microbiota often reflects the characteristics of the local environment [24, 56] and migratory birds may acquire as much as 25% of the gut microbiota from environmental sources [57]. In this study, we found evidence supporting environmental acquisition of microorganisms in the gut microbiota of white-tailed eagles. For instance, the genus *Plesiomonas*, which was commonly found in fresh surface water marine environments [58], was significantly elevated during autumn migration of white-tailed eagles but was nearly absent in most individuals during the spring migration. Conversely, the genus *Acinetobacter*, which was reportedly prevalent in soil and water [59], was enriched in the spring migration and rarely detected during the autumn migration of white-tailed eagles.

White-tailed eagles migrating through the Jingxin Wetland follow the EAAF, which is characterized by extensive oceanic crossings that distinguish it from the African-Eurasian and American flyways. During the autumn migration, white-tailed eagles travel from their breeding grounds on the Kamchatka Peninsula, crossing the Sea of Okhotsk before reaching the Jingxin Wetland. Whereas in the spring migration, they traverse the Sea of Japan en route to the wetland. Therefore, we hypothesize that *Plesiomonas* and *Acinetobacter* may originate from distinct environmental sources encountered along the migratory pathways. Specifically, *Plesiomonas* likely originated from the Sea of Okhotsk during the autumn migration, whereas *Acinetobacter* might be derived from the Sea of Japan during the spring migration. These microorganisms might have been ingested by white-tailed eagles through their diet [60]. However, we cannot rule out the possibility that these differences might also stem from temporal and spatial variations in the environmental microbiota of the Jingxin Wetland itself, as avian microbiota can reflect environmental changes including exposure to new environments, within 24–48 h [6, 61].

### Diet

We observed an increase in alpha diversity in the spring migration season compared to the autumn season (Sobs, but not Chao1), which may be due to the increased availability of food resources like fish and waterfowl in the spring as the ice of the Jingxin Wetland began to melt in March. A more diverse diet has been found to associate with higher gut microbiome diversity [62], which can potentially improve the stability of the gut microbiome and benefit the host. Generally, a more diverse gut microbiota is more stable because functionally similar taxa can potentially replace one another and therefore, the host is more tolerant to changes in the gut microbiome [63]. Also, as the gut microbiome is involved in, for example, host metabolism and digestion by breaking down dietary items into compounds that can be used by the host, a diverse gut microbiome can influence host nutritional uptake and physiology. It’s worth mentioning that pathogen *Escherichia-Shigella*, which was proved negatively correlated to body condition [54], was found enriched in the autumn migration group.

During the autumn migration, there was an upward trend in the prevalence of Campilobacterota, which includes *Campylobacter*, a primary pathogen responsible for one of the most commonly reported foodborne infections [64]. Poultry are recognized as the major reservoir of *Campylobacter* [65], and the consumption of contaminated chicken meat is considered the leading cause of human Campylobacteriosis. It’s reported that some birdwatchers in the Jingxin Wetland feed white-tailed eagles with chicken carcasses, some of which might be partially contaminated, potentially contributing to the elevated levels of Campilobacterota. Although the pathogenicity of *Campylobacter* in birds remains unclear, the vast distance migration may facilitate the spread of this zoonotic pathogen, underscoring the need for vigilant monitoring and proactive strategies to prevent potential outbreaks.

The increased abundance of the genera *Clostridium_sensu_stricto_13* and *Brochothrix* during the spring migration suggests potential shifts in microbial composition associated with dietary changes. Members of *Clostridium_sensu_stricto_13* and *Brochothrix* have been previously linked to the decomposition of protein-rich food sources, such as carrion and meat. While our sequencing approach does not allow for species-level identification, the presence of these genera aligns with the dietary habits of white-tailed eagles, which are known to scavenge more frequently during certain seasons. Further functional analysis, such as metagenomic sequencing, would be necessary to confirm the specific roles of these microbial taxa. As a raptor, the white-tailed eagle predominantly consumes fish and meat [66], which are often nutrient-rich environments that support the growth of *C. estertheticum* and *B. thermosphacta*. *C. estertheticum* and *B. thermosphacta* are well known for their role in the spoilage of meat, particularly under cold and low-oxygen conditions [67]. The high abundance of these two gut microbes observed during the spring migration may reflect the white-tailed eagle’s consumption of carrion during this period, aligning with previous studies on its dietary habits [68].

Future studies should adopt more precise sampling methods, such as utilizing artificial intelligence for individual bird identification, to reduce potential biases caused by differences in sex and age composition [69, 70]. Additionally, previous studies have reported significant variations in gut microbiota across different ages and sexes in birds [71, 72], suggesting that the seasonal differences observed in this study may be partially influenced by sex- and age-related factors. Furthermore, this study is based on 16S rRNA sequencing, which allows for the identification of seasonal variations in gut microbiota but does not provide strain-level resolution or functional insights into detected pathogens. As a result, our findings are limited to reporting the relative abundance of potential pathogens without assessing their pathogenicity or specific impacts on raptor health. Future research should incorporate metagenomics, functional gene analysis (e.g., qPCR), and host immune response assessments to better evaluate the potential role of these microorganisms in host health and ecosystem dynamics. Moreover, our study was conducted at a single stopover site, and environmental factors may have influenced microbiota composition. Therefore, comparative studies across multiple stopover sites and wintering grounds would be beneficial in further understanding the role of environmental factors in shaping the gut microbiota of migratory raptors.

## Conclusions

In this study, we compared the gut microbiota of white-tailed eagles during the autumn and the spring migration at a key stopover site, aiming to understand seasonal variations and infer the factors that might drive these changes. Our results reveal that the white-tailed eagle gut microbiota is primarily dominated by Firmicutes, consistent with previous studies on other avian species. However, significant seasonal shifts in microbiome composition were observed, likely reflecting differences in migration, diet and environmental exposure. Notably, several bacterial genera with known pathogenic potential, including *Fusobacterium* and *Escherichia-Shigella*, were enriched during the autumn migration, potentially indicating higher pathogen susceptibility. Individuals in the spring migration group showed elevated diversity and an increased abundance of *Clostridium sensu stricto 13* and *Brochothrix*, likely due to the white-tailed eagle’s consumption of carrion. The presence of differentially abundant taxa commonly acquired from the environment, such as *Plesiomonas* and *Acinetobacter*, suggests that habitat differences between migration seasons may contribute to the observed microbiome variations. These findings provide insights into how migration season influences gut microbiota composition in migratory raptors, with potential implications for disease transmission dynamics. Future research will delve deeper into the ecological and health implications of these findings for migratory raptors.

## Methods

### Samples collection

**Fig. 6:**
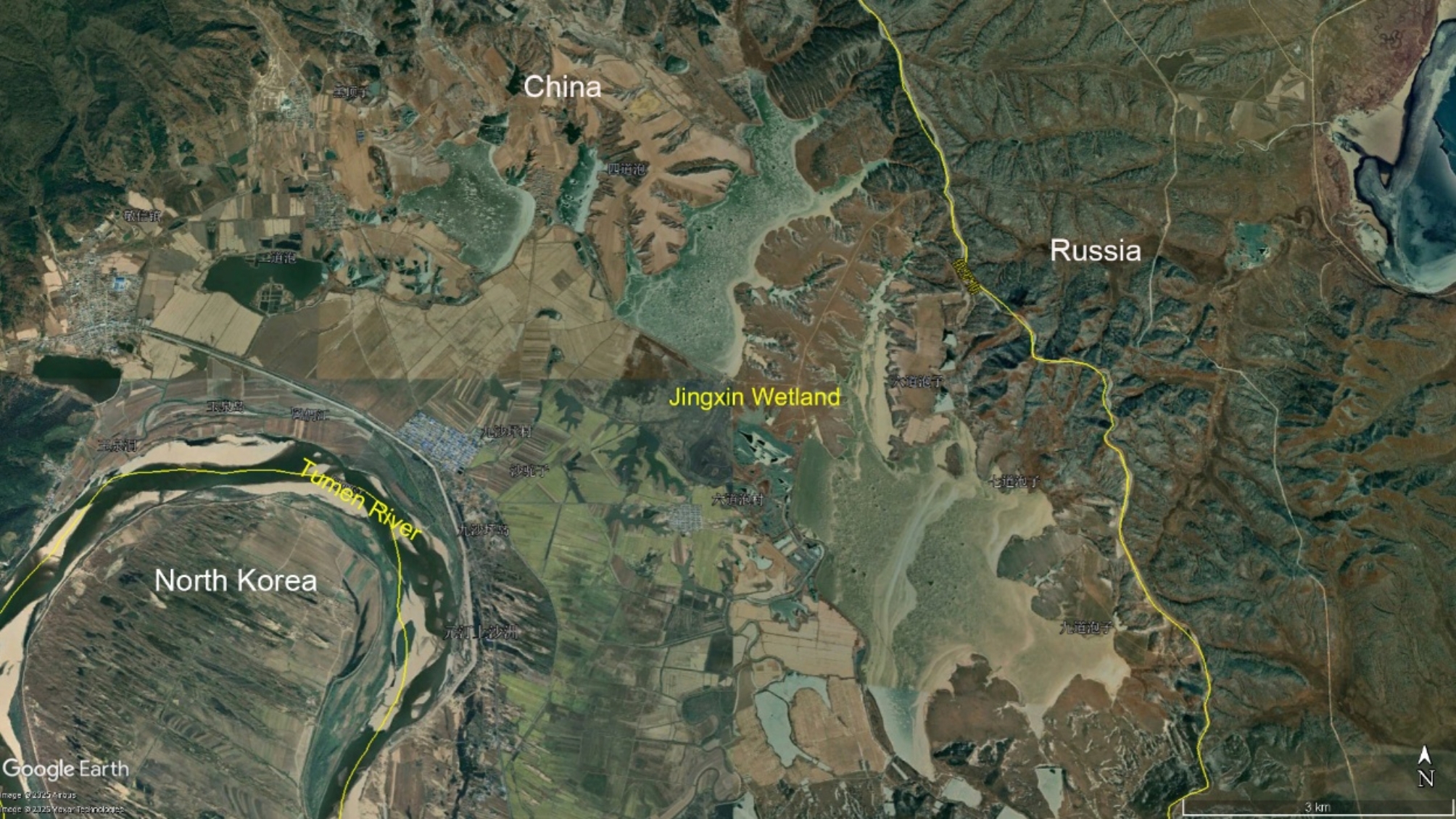
Study Area Distribution: Jingxin Wetland and Surrounding Regions

A total of 33 fecal samples were collected in the Jingxin Wetland (130°25′ − 130°39′N, 42°27′ − 42°40′E), a critical stopover site for white-tailed eagles located in the northeast China (Fig. 6). Specifically, 19 samples were collected during the autumn migration period in December, 2021 while 14 samples were collected during the spring migration period in March, 2022. Only fresh fecal samples from individual excretions, free from visible contamination, were collected. We employed a non-invasive sampling method, where fecal samples were collected only after individuals had excreted naturally, ensuring minimal disturbance to the birds. No individuals were captured for sampling, and therefore, data such as ringing, sexing, or body condition were not recorded. All sampling tools were sterilized to prevent cross-contamination. Samples were stored individually in sterile disposable sealed bags and placed in an ice box. The samples were immediately transported to the laboratory and stored at -80 °C to ensure their integrity for subsequent DNA extraction.

### DNA extraction, PCR amplification and High-throughput sequencing

DNA was isolated from the fecal samples of white-tailed eagles using the QIAamp Stool Mini Kit (51504) (Qiagen, Germany) in accordance with the manufacturer’s guidelines. The extracted DNA quality was evaluated using a Nanodrop 2000. Library preparation involved amplifying the V3 and V4 regions of the 16S rRNA gene, employing the primers 338F (5′-barcode-ACTCCTACGGGAGGCAGCAG-3′) and 806R (5′-GGACTACHVGGGTWTCTAAT-3′) [73]. The PCR conditions included an initial denaturation step at 95 °C for 3 min, followed by 29 cycles consisting of denaturation at 95 °C for 30 s, annealing at 53 °C for 30 s, and extension at 72 °C for 45 s, with a final elongation at 72 °C for 10 min. Each PCR reaction was conducted in triplicate, with a reaction mixture of 20 µL containing 4 µL of 5× FastPfu Buffer, 2 µL of 2.5 mM dNTPs, 0.8 µL of each primer (5 µM), 0.4 µL of FastPfu Polymerase, 0.2 µL of BSA, and 10 ng of template DNA.

PCR products were separated on 2% agarose gels, extracted, and purified using the AxyPrep DNA Gel Extraction kit (Axygen Biosciences, Union City, CA, USA), following the provided protocols. The purified products were quantified with the QuantiFluor™-ST system (Promega, Madison, WI, USA). Equimolar amounts of the purified products were pooled, and high-throughput sequencing was carried out on an Illumina MiSeq PE300 platform, following the standard protocols (Shanghai Majorbio Bio-pharm Technology Co., Ltd, Shanghai, China).

The data were analyzed using the free online platform of Majorbio Cloud Platform (www.majorbio.com).

### Statistical analysis

We processed raw sequence data with the quantitative insights into microbial ecology (QIIME2 version 2020.2) pipeline [74].

Raw files were demultiplexed and quality-filtered using QIIME2 (version 2020.2) [74]. The sequences were clustered into operational taxonomic units (OTUs) at 97% similarity using UPARSE (version 7.0). The taxonomy of 16S rRNA sequences was analyzed using the RDP Classifier1 (http://rdp.cme.msu.edu/) against the Silva (SSU 138) 16S rRNA database with a confidence threshold of 70%.

For the visualization of microbiota overlap, Venn diagrams were generated using R (version 3.3.1) to illustrate the shared and unique microbial taxa between groups. Alpha diversity indices were calculated and generated using mothur (version v.1.30.2) and differences in alpha diversity between groups were assessed using the Wilcoxon rank-sum test performed in R utilizing the “boot” (version 1.3.18) and “stats” (version 3.3.1) packages. To standardize everything, we randomly selected the size of the smallest library sequences from each sample 1000 times and calculate the average. To visualize changes in microbiota communities, the distance matrix of beta diversity was calculated using QIIME (2020.2.0) and non-metric multidimensional scaling (NMDS) analysis based on unweighted UniFrac was performed and displayed using “vegan” package (version 2.4.3) in R. Similarly, the principal coordinate analysis (PCoA) based on Binary Jaccard distances was generated by R. To evaluate whether microbial community structures significantly differed between groups, ANOSIM (Analysis of Similarities) was conducted based on 999 permutations in association with both PCoA and NMDS ordinations. To analyze whether there were differences in microbial composition between the two groups and to identify significantly different microbial taxa, we conducted a significance test for group differences using the Wilcoxon rank-sum test. Significance was considered at a threshold of *p* < 0.05 for all tests.

## Electronic supplementary material

Below is the link to the electronic supplementary material.

Supplementary Material 1

## Data Availability

The data availability information is as follows: The raw data can be accessed from the NCBI Sequence Read Archive (SRA) under the accession number PRJNA1198865.

## Declarations

### Open access

I confirm that I understand Animal Microbiome is an open access journal that levies an article processing charge per articles accepted for publication. By submitting my article I agree to pay this charge in full if my article is accepted for publication.

### Dual publication

The results/data/figures in this manuscript have not been published elsewhere, nor are they under consideration (from you or one of your Contributing Authors) by another publisher.

### Third party material

All of the material is owned by the authors and/or no permissions are required.
