# Supplementary material for: Seasonal variation in gut microbiota of migratory wild raptors: a case study in white-tailed eagles
Source: Anim Microbiome. 2025 Apr 17;7:37. doi: 10.1186/s42523-025-00406-y (PMC12007228; doi:10.1186/s42523-025-00406-y)
Supplement: Supplementary file 1 — Supplementary Material 1 [file 42523_2025_406_MOESM1_ESM.docx]

**Fig S1.** Rarefaction Curves

**Fig S2.** The bar plot of relative abundance in the gut microbiota of white-tailed eagles at phylum (**A**) and genus (**B**) level. A means the autumn migration group while S means spring migration group.
